# An integrated multi-study analysis of intra-subject variability in cerebrospinal fluid amyloid-β concentrations collected by lumbar puncture and indwelling lumbar catheter

**DOI:** 10.1186/s13195-015-0136-z

**Published:** 2015-07-29

**Authors:** Brendan P. Lucey, Celedon Gonzales, Ujjwas Das, Jinhe Li, Eric R. Siemers, J. Randall Slemmon, Randall J. Bateman, Yafei Huang, Gerard B. Fox, Jurgen A.H.R. Claassen, Diane Slats, Marcel M. Verbeek, Gary Tong, Holly Soares, Mary J. Savage, Matthew Kennedy, Mark Forman, Magnus Sjögren, Richard Margolin, Xia Chen, Martin R. Farlow, Robert A. Dean, Jeffrey F. Waring

**Affiliations:** Department of Neurology, Washington University School of Medicine, Campus Box 8111, 660 South Euclid Avenue, St Louis, MO 63110 USA; Hope Center for Neurological Disorders, Department of Neurology, Washington University School of Medicine, Campus Box 8111, 660 South Euclid Avenue, St Louis, MO 63110 USA; Eli Lilly and Company, Lilly Corporate Center, 893 South Delaware Avenue, Indianapolis, IN 46285 USA; AbbVie Inc., 1 N. Waukegan Road, North Chicago, IL 6004 USA; Johnson and Johnson, One Johnson & Johnson Plaza, New Brunswick, NJ 08933 USA; Department of Medicine, St. Luke’s Hospital, 232 South Woodsmill Road, Chesterfield, MO 63017 USA; Department of Geriatric Medicine, Donders Institute for Brain, Cognition, and Behaviour, Radboud Alzheimer Center, Radboud University Medical Center, Route 925, PO Box 9101, 6500 HB Nijmegen, The Netherlands; Department of Neurology, 830 TML, Neurochemistry Lab, Radboud University Nijmegen Medical Center, PO Box 9101, 6500 HB Nijmegen, The Netherlands; Lundbeck LLC, Four Parkway North, Deerfield, IL 60015 USA; Bristol-Myers Squibb, 311 Pennington-Rocky Hill Road, Pennington, NJ 08534 USA; Merck and Company, RY50-1D-131, 126 East Lincoln Avenue, PO Box 2000, Rahway, NJ 07065 USA; Merck and Company, 33 Avenue Louis Pasteur, Boston, MA 02115 USA; Merck and Company, 2000 Galloping Hill Road, Kenilworth, NJ 07033 USA; Mental Health Centre Ballerup, Capital Region of Denmark, Maglevanget 2, 2750, Ballerup, Denmark; CereSpir, Inc., 41 Madison Avenue, 31st Floor, New York, NY 10010 USA; Boeringher Ingelheim, 900 Ridgebury Road, Ridgefield, CT 06877 USA; Department of Neurology, Indiana University School of Medicine, Goodman Hall, Suite 4700, 355 West 16th Street, Indianapolis, IN 46202 USA

## Abstract

**Introduction:**

Amyloid-β (Aβ) has been investigated as a diagnostic biomarker and therapeutic drug target. Recent studies found that cerebrospinal fluid (CSF) Aβ fluctuates over time, including as a diurnal pattern, and increases in absolute concentration with serial collection. It is currently unknown what effect differences in CSF collection methodology have on Aβ variability. In this study, we sought to determine the effect of different collection methodologies on the stability of CSF Aβ concentrations over time.

**Methods:**

Grouped analysis of CSF Aβ levels from multiple industry and academic groups collected by either lumbar puncture (n=83) or indwelling lumbar catheter (n=178). Participants were either placebo or untreated subjects from clinical drug trials or observational studies. Participants had CSF collected by lumbar puncture or lumbar catheter for quantitation of Aβ concentration by enzyme linked immunosorbent assay. Data from all sponsors was converted to percent of the mean for Aβ40 and Aβ42 for comparison. Repeated measures analysis of variance was performed to assess for factors affecting the linear rise of Aβ concentrations over time.

**Results:**

Analysis of studies collecting CSF via lumbar catheter revealed tremendous inter-subject variability of Aβ40 and Aβ42 as well as an Aβ diurnal pattern in all of the sponsors’ studies. In contrast, Aβ concentrations from CSF samples collected at two time points by lumbar puncture showed no significant differences. Repeated measures analysis of variance found that only time and draw frequency were significantly associated with the slope of linear rise in Aβ40 and Aβ42 concentrations during the first 6 hours of collection.

**Conclusions:**

Based on our findings, we recommend minimizing the frequency of CSF draws in studies measuring Aβ levels and keeping the frequency standardized between experimental groups. The Aβ diurnal pattern was noted in all sponsors’ studies and was not an artifact of study design. Averaging Aβ concentrations at each time point is recommended to minimize the effect of individual variability. Indwelling lumbar catheters are an invaluable research tool for following changes in CSF Aβ over 24-48 hours, but factors affecting Aβ concentration such as linear rise and diurnal variation need to be accounted for in planning study designs.

**Electronic supplementary material:**

The online version of this article (doi:10.1186/s13195-015-0136-z) contains supplementary material, which is available to authorized users.

## Introduction

Alzheimer’s disease (AD) is a neurodegenerative disorder characterized by degeneration of neurons and their synapses leading to progressive cognitive impairment. In the United States, AD is estimated to be the third leading cause of death [1] and to have a financial burden on society comparable with heart disease and cancer [2]. The hallmark of AD at the microscopic level is an overabundance in the brain of extracellular plaques formed by abnormally folded amyloid-beta (Aβ) and intracellular neurofibrillary tangles of tau. The amyloid hypothesis proposes that the deposition of Aβ in the brain is a key first step in AD pathogenesis that precedes the onset of clinical symptoms by many years [3, 4]. Therefore, Aβ has been investigated both as a diagnostic biomarker for amyloid deposition, measured by imaging (e.g. positron emission tomography imaging with Pittsburgh Compound B (PiB-PET)) or Aβ42 concentration in the cerebrospinal fluid (CSF) [5], and as a potential therapeutic target [6, 7].

Previous studies measured Aβ concentrations before and after amyloid deposition to understand potential changes in Aβ metabolism during AD pathogenesis. In these studies, CSF was collected from individuals infrequently via lumbar puncture, generally limited to the beginning and the end of the study. The importance of understanding Aβ metabolism for pharmacologic modeling during trials of investigational compounds led to clinical studies collecting CSF samples repeatedly over 24–48 hours via an indwelling lumbar catheter. A number of these studies have demonstrated considerable intra-subject variability in the levels of CSF Aβ [8, 9] and have also shown a diurnal fluctuation in CSF Aβ that follows the sleep–wake cycle [10]. This diurnal oscillation has also been noted in plasma [11]. The frequency and amount of CSF collected vary greatly in studies employing indwelling catheters. Differences in CSF collection methods could be a factor contributing to the observed variability of Aβ levels. For instance, a recent study found that CSF sampling frequencies and/or sampling volume contributes to intra-subject variability in CSF Aβ levels [12]. Further, sampling hourly via a lumbar catheter has been reported to result in a progressive linear rise in Aβ concentrations. The cause of the Aβ linear rise is unknown but is suspected to be due to changes in CSF flow [10].

Understanding how different collection methodologies affect the stability of CSF Aβ levels over time is of paramount importance for the design of clinical trials, where these biomarkers would be utilized to study pharmacodynamic activity, and ultimately may determine whether Aβ has utility in a diagnostic fashion. Several investigational compounds that target Aβ are currently in clinical trials [13]. In many cases, particularly in phase I and early phase II trials, the levels of Aβ in CSF are monitored to assess target engagement. Both lumbar punctures and lumbar catheters are used during clinical trials to collect CSF. For instance, both plasma and CSF Aβ levels were monitored in volunteers following treatment with a gamma-secretase inhibitor [14, 15]. In these studies, the levels of plasma Aβ were reduced in a dose-responsive manner, but no significant decreases were observed in the levels of CSF Aβ. In addition, a recent study examined the levels of CSF Aβ in AD subjects receiving bapinuzimab, an antibody that targets Aβ. No changes in the levels of CSF Aβ from baseline were observed following treatment, although there was a decrease in the levels of CSF tau [16]. In contrast, subjects treated with solanezumab, a selective antibody that targets soluble Aβ, demonstrated a dose-dependent increase in total CSF levels of Aβ40 and Aβ42 [17]. This increase may be due to downstream effects on Aβ plaque dissolution. All of these studies collected CSF via lumbar puncture. Lumbar catheters have also been used for serial CSF sampling, such as when tracking changes in Aβ kinetics (i.e. production and clearance) using stable isotope labeling kinetics [18]. As more therapies targeting Aβ advance into clinical trials, it is expected that the use of Aβ and other CSF biomarkers will increase substantially [19].

To identify the most relevant factors that may contribute to intra-subject variability in CSF Aβ levels, and as part of the Alzheimer’s Disease Neuroimaging Initiative (ADNI) consortia, we shared data from multiple clinical studies on CSF Aβ measurements from placebo or untreated subjects and conducted a meta-analysis to identify factors that may result in changes in CSF Aβ levels. The combined dataset is the largest collection of participants with serial CSF sampling via a lumbar catheter (*n* = 178). Our results suggest that the slope of the linear rise in CSF Aβ levels is dependent on the sampling frequency, but does not affect the hourly variability manifesting as an Aβ diurnal pattern. The results from this study will help guide clinical trial design, and provide insight into the relative stability of proteins associated with AD in CSF.

## Materials and methods

Each of the following pharmaceutical research companies or academic research centers contributed the CSF results for Aβ isoform x-40 and x-42 concentrations from assays developed commercially or within the research centers: Bristol-Myers Squibb (BMS, New York, NY, USA), AbbVie Inc. (North Chicago, IL, USA), Eli Lilly and Company (Indianapolis, IN, USA), Merck and Company (Kenilworth, NJ, USA), Radboud University Medical Center (Nijmegen, the Netherlands), and Washington University (St Louis, MO, USA). All of the samples were obtained via either lumbar puncture or serial sampling through an indwelling lumbar catheter. Samples were obtained from subjects who participated in clinical research studies. Only participants who were given placebo treatment in pharmacological trials were included in this study. Table 1 presents information about the lumbar studies, while Table 2 presents detailed information about each study and the demographic information available for the indwelling catheter studies. Whether or not study subjects were negative or positive for amyloidosis was known for only one study, and therefore this variable is not addressed in the analysis. Unless otherwise noted, the timing of lumbar puncture or lumbar catheter placement was standardized within a study. Sleep–wake activity was not monitored before, during, or after CSF collection.

**Table 1:** Lumbar puncture studies

Sponsor	Study	Subjects (*n*)	Type of subjects	Age (mean (SD))	CSF sample volume per draw (ml)
Lilly	L1	19	9 AD, 10 healthy	Not obtained	Not obtained
	L2	10	Healthy	61 (8)	16 ml per draw
	L3	34	AD	71.7 (6.4)	10 ml per draw
	L4	13	AD	69 (10)	25 ml per draw
	L5-A	7	Healthy	52 (6)	Not obtained

*AD* Alzheimer’s disease, *CSF* cerebrospinal fluid, *Lilly* Eli Lilly and Company (Indianapolis, IN, USA), *SD* standard deviation

**Table 2:** In-dwelling catheter studies

Sponsor	Study	Subjects (*n*)	Study site	Gender (male/female)	Age (mean (SD))	CSF sampling volume/frequency
AbbVie	A1	6	CCT	6/10	38.2 (9)	8 ml approximately every 2 hours for 24 hours
	A2	22	CCT	17/5	68.5 (7.2)	8 ml approximately every 4 hours for 24 hours
	A3	8	CCT	8/0	35.3 (8.1)	8 ml approximately every 1 hour for 24 hours; participants repeated study
BMS	B1	10	CCT	10/0	31.6 (6.5)	7 ml every hour for 48 hours
Lilly	L6	6	LCRU		60.8 (8.4)	0.1 ml every 1 minute
	L7	7	WU	7/0	34 (7)	6 ml every hour for 36 hours
	L8	10	PAREXEL		34.6 (8)	Volume not obtained; every 1 hour for 10 hours; every 2 hours for 26 hours
	L5-B	2	LCRU		52.7 (4)	Volume not obtained; every 1 hour for 10 hours
Merck	M1	6	VU	3/3	37.3 (4.6)	0.5 ml every 5 minutes
	M2	3	CCT	3/0	26 (7)	6 ml every 1 hour for 8 hours; every 2 hours for 4 hours; after the last 4 hours
	M3	6	CCT	4/2	54.3 (7.9)	4.5 ml every 1 hour for 16 hours; every 2 hours for 10 hours
	M4	9	CCT	6/2^a^	52 (7.8)^a^	8 ml every 1 hour for 14 hours; every 2 hours for 12 hours
	M5	6	CCT	6/0	38.3 (6)	6 ml every 1 hour for 14 hours; every 2 hours for 12 hours
	M6	4	SGS	3/1	56.5 (10)	Same as M5
	M7	4	SGS	2/2	46.5 (10)	Same as M5
RUMC	R1	12	RUMC		70.3 (7.5)	6 ml every 1 hour for 36 hours
WU	W1	57	WU	29/28	60.7 (19)	6 ml every 1 hour for 36 hours
Total		178				

^a^Missing gender and age for one participant

*AbbVie* AbbVie Inc. (North Chicago, IL, USA), *AD* Alzheimer’s disease, *BMS* Bristol-Myers Squibb (New York, NY, USA), *CCT* California Clinical Trials Medical Group (Glendale, CA, USA), *CSF* cerebrospinal fluid, *LCRU* Lilly Clinical Research Unit (Indianapolis, IN, USA), *Lilly* Eli Lilly and Company (Indianapolis, IN, USA), *Merck* Merck Research Labs (Merck and Company (Kenilworth, NJ, USA); Boston, MA, USA; Upper Gwynedd, PA, USA), *Parexel* PAREXEL International Early Phase (Los Angeles, CA, USA), *RUMC* Radboud University Medical Center (Nijmegen, the Netherlands), *SD* standard deviation, *SGS* Clinical Research (Antwerpen, Belgium), *VU* Vanderbilt University (Nashville, TN, USA), *WU* Washington University (St Louis, MO, USA)

### Methods from Bristol-Myers Squibb serial sampling studies

#### Subjects and dosing

The clinical phase of the study was conducted from December 2007 to March 2008 at the California Clinical Trials Medical Group (Glendale, CA, USA). Healthy male subjects between 20 and 45 years of age were enrolled in the study. This study was conducted using Good Clinical Practice, as defined by the International Conference on Harmonization, and in compliance with the Declaration of Helsinki, and was approved by the Institutional Review Board of the California Clinical Trials Medical Group where the study was conducted. All subjects provided written informed consent prior to study entry.

#### Sample collection and assay procedures

For repeated CSF sampling, a 19G Dura-Flex PLUS epidural lumbar catheter (Smiths Medical, Saint Paul, MN, USA) was placed at the L3/L4 interspace of each subject approximately 3 hours before dosing. Four CSF samples of approximately 7–10 ml each were collected in Falcon polypropylene tubes (BD Biosciences, Franklin Lakes, NJ, USA) within 2 hours before placebo administration starting at approximately 7:00 a.m., and then at hourly intervals for up to 41 hours after dosing. The start time of CSF collection for all participants was approximately between 6:30 and 7:30 a.m. Samples of CSF were immediately frozen and stored at or below –80 °C until shipment to the analytical laboratory (Bristol-Myers Squibb Bioanalytical Sciences, Princeton, NJ, USA) on dry ice. Aβ peptide concentrations were determined using MS6000 human (6E10) Aβ 3-plex kits (Meso Scale Discovery, Gaithersburg, MD, USA). Samples were analyzed for concentrations of Aβ38, Aβ40, and Aβ42. The between-run and within-run coefficient of variation (CV) for the analytical quality control checks (QCs) for Aβ38 and Aβ40 concentrations was <25 %, whereas the between-run CV for the analytical QCs for Aβ42 concentration was >30 %. QCs from Aβ42 were therefore only evaluated within run and accepted when the within-run CV was <20 %. All samples for a subject were analyzed in the same plate or on the same day to minimize the variability of Aβ42 assessments. In addition, CSF Aβ42 levels were measured using high-performance liquid chromatography (HPLC) as described previously [20].

### Methods from AbbVie serial sampling studies

#### Subjects and dosing

The three AbbVie studies were all conducted at PAREXEL/California Clinical Trials (Glendale, CA, USA). All three studies were conducted in compliance with the Declaration of Helsinki and the International Conference on Harmonization for Good Clinical Practice guidelines. Institutional review board approval was obtained from PAREXEL for all three studies and written informed consent was obtained from all subjects in all three studies. All subjects were screened to be in good general health and without neurologic disease. A lumbar catheter was placed immediately before CSF sample collection started. The subjects were encouraged to stay in bed, and were allowed free choice of when to sleep throughout the study. Each study was conducted on a different group of subjects and each with a different CSF sampling procedure. There were no subject drop-outs or refusals. In Study A1, six healthy young men (25–47 years old) participated, and 5 ml CSF and 4 ml blood were collected from each subject at 0 (6:00 a.m.), 1, 3, 5, 6, 7, 8, 10, 12, 14, 18, 22, and 26 hours. In Study A2, five healthy older men, two healthy older women (61–74 years old), and 15 subjects diagnosed with AD (58–83 years old) participated. Seven milliliters of CSF and 10 ml blood were collected from each subject at 0 (5:30 a.m.), 1, 4, 8, 12, 18, and 24 hours. In Study A3, eight healthy young men (24–45 years old) participated and were divided into two groups. In one group of four subjects, 6 ml CSF and 4 ml blood were collected from each subject at 18 time points over 24 hours (“higher frequency” period) at 0 (8:00 a.m.), 1, 2, 3, 4, 5, 6, 7, 8, 9, 10, 12, 14, 16, 18, 20, 22, and 24 hours. Ten days later, CSF was collected from the same four subjects at seven time points over 24 hours (“lower frequency” period) at 0 (8:00 a.m.), 1, 4, 8, 12, 18, and 24 hours. In another group of four subjects, CSF was first collected at lower frequency, followed by higher frequency CSF collection 10 days later from the same subjects. During each CSF collection in these three studies, the first 2 ml (corresponding to the tubing dead space) were voided. CSF aliquots of 250 μl were frozen at –80 °C immediately after collection in 1 ml siliconized polypropylene tubes.

#### Sample collection and assay procedures

CSF Aβ38, Aβ40, and Aβ42 concentrations were measured using human Aβ38/Aβ40/Aβ42 multiplex assays (using 6E10 as detection antibody) following the manufacturer’s procedure (Meso Scale Discovery, Gaithersburg, MD). To avoid inter-plate variation, all samples from each subject were measured together in duplicates on the same plate, and all of the samples from the same study were measured on the same day. To avoid bias due to intra-plate variation, all samples from each subject were randomized on the plate. The means of the intra-plate CVs for the duplicate samples including a control human CSF sample were <10 %. The means of the inter-plate CVs for the control human CSF sample were <10 %.

### Methods from Lilly lumbar puncture and serial sampling studies

#### Subjects and dosing

Eight clinical studies involving CSF sampling for analysis of Aβ peptides were sponsored by Eli Lilly and Company. Details of collections are presented in Tables 1 and 2. Each study underwent institutional review board approval and was conducted in compliance with the Declaration of Helsinki and the International Conference on Harmonization for Good Clinical Practice guidelines. Healthy subjects participating in each study provided written informed consent. In studies involving patients with probable AD, written informed consent was obtained from the subject as well as their legally authorized representative and their primary caregiver. The diagnosis of probable AD was based on the National Institute of Neurological and Communicative Disorders and Stroke and the Alzheimer’s Disease and Related Disorders Association guidelines [21]. Subjects were required to have a Mini-Mental State Examination (MMSE) score between 14 and 26 inclusive [22], a Modified Hachinski Ischemic Index score ≤4 [23], and central nervous system imaging of the brain by computerized tomography scan or magnetic resonance imaging (MRI) compatible with AD within the past 12 months.

#### Lilly lumbar puncture studies

Lilly Study 1 was conducted at Indiana University (Indianapolis, IN, USA) in compliance with the Declaration of Helsinki and the International Conference on Harmonization for Good Clinical Practice guidelines. Institutional review board approval was obtained from Indiana University and written informed consent was obtained from all subjects. The study was designed to define within-subject variability of CSF Aβ isoforms from two lumbar punctures taken 1–2 weeks apart from 10 AD patients and nine healthy, age-matched volunteers 45 years of age or older (ages ranged from 21 to 84 years old) in the absence of any treatment [24]. Subjects were ambulatory for 2 hours prior to CSF collection, which occurred at 2:00 p.m. ± 1 hour. Collected CSF was immediately aliquoted into polypropylene storage tubes, quick frozen, and maintained at –70 °C.

Lilly Study 2 **was** conducted at the Lilly Clinical Research Unit (Indianapolis, IN, USA) in compliance with the Declaration of Helsinki and the International Conference on Harmonization for Good Clinical Practice guidelines. Institutional review board approval was obtained from Indiana University and written informed consent was obtained from all subjects. The study was a single-blind, placebo-controlled, multiple-dose, dose-escalation study of LY450139 conducted in a combined inpatient/outpatient fashion [14]. Ten healthy male and postmenopausal or surgically infertile female volunteers over 45 years of age (mean ± standard deviation (SD) = 61 ± 8) underwent lumbar puncture using a 25-gauge needle for CSF sample collection prior to initiation of and 6 hours following 14 ± 1 days of single daily oral administration of a placebo treatment. Subjects were ambulatory (nonrecumbent) for at least 2 hours prior to each lumbar puncture. A total of 16 ml CSF was incrementally collected as 4 × 4 ml. Each fraction was subsequently aliquoted into polypropylene cryovials and immediately frozen. Aliquots were maintained frozen at –70 °C.

Lilly Study 3 was approved by the institutional review board for each participating site (Oregon Health and Science University, Portland, OR, USA; Indiana University, Indianapolis, IN, USA; University of Rochester, Rochester, NY, USA; California Clinical Trials, Glendale, CA, USA; Washington University, St Louis, MO, USA; Mayo Clinic, Rochester, MN, USA). The protocol conformed to the International Conference on Harmonization guidelines and to the Declaration of Helsinki. Written informed consent was obtained from all subjects. The study was a multisite randomized, double-blind, placebo-controlled trial also involving LY450139 [25]. Thirty-four subjects (22 male and 12 postmenopausal female) 50 years or older (mean age ± SD = 71.7 ± 6.4) with probable mild to moderate AD underwent lumbar puncture for collection of CSF prior to and approximately 6 weeks following daily oral placebo administration. CSF was collected approximately 4 hours following the last dose of placebo. Subjects were ambulatory (nonrecumbent) for at least 2 hours prior to each lumbar puncture. Approximately 10 ml CSF was taken at approximately the same time of day. CSF was collected, mixed by inversion and aliquoted in polypropylene tubes, frozen, and maintained –70 °C until analyzed.

Lilly Study 4 was approved by the institutional review board for each participating site (University of California (San Diego), San Diego, CA, USA; University of Pennsylvania, Philadelphia, PA, USA; Oregon Health and Science University, Portland, OR, USA; Indiana University, Indianapolis, IN, USA; Washington University, St Louis, MO, USA; University of Washington, Seattle, WA, USA; Georgetown University, Washington, DC, USA). The protocol conformed to the International Conference on Harmonization guidelines and to the Declaration of Helsinki. Written informed consent was obtained from all subjects. The study was a multisite, randomized, parallel-arm, placebo-controlled, dose-escalation trial of LY450139 in mild to moderate AD patients 50 years of age or older (mean ± SD = 69 ± 10) with a treatment duration of approximately 14 weeks [15]. Thirteen of 15 male and postmenopausal female subjects were randomized to an oral placebo treatment and completed the study, including successful lumbar puncture for CSF collection prior to and at the end of the treatment period. Subjects were ambulatory (nonrecumbent) for at least 2 hours prior to each lumbar puncture in which 25 ml CSF were obtained at approximately the same time of day. Results from these 13 individuals are included in the present investigation. CSF was collected into chilled, polypropylene tubes, rapidly frozen, and maintained at –70 °C until analyzed.

Lilly Study 5-A and Study 5-B were conducted at the Lilly Research Laboratories Clinic (Indianapolis, IN, USA). Institutional review board approval was obtained from Indiana University. The protocol conformed to the International Conference on Harmonization guidelines and to the Declaration of Helsinki. Written informed consent was obtained from all subjects. Study 5-A and Study 5-B were two parts of a double-blind, placebo-controlled, single ascending dose study of LY450139 [26]. In part A, seven healthy male and female volunteers 40 years and older (mean ± SD = 51 ± 5.7) underwent two lumbar punctures separated by approximately 3 weeks to obtain CSF 4 hours following oral administration of placebo. In part B, two healthy subjects participating in part A underwent indwelling subarachnoid catheterization approximately 3 weeks after initiating daily placebo administration to permit serial CSF sampling for 10–12 hours following the last dose of placebo.

#### Lilly serial sampling studies

Lilly Study 6 was conducted at the Lilly Clinical Research Unit. Institutional review board approval was obtained from Indiana University. The protocol conformed to the International Conference on Harmonization guidelines and to the Declaration of Helsinki. Written informed consent was obtained from all subjects. The study was a placebo-controlled, single-dose, pharmacokinetic/pharmacodynamic study of LY450139 in mild to moderate AD patients and healthy volunteers [27]. Results from six healthy volunteer subjects over the age of 45 are included in the present investigation. A single oral dose of placebo was administered between 9:00 a.m. and 1:00 p.m., approximately 30 minutes after placement of a sterile indwelling subarachnoid catheter introduced through the L4/L5 lumbar space and connected to approximately 200 cm of sterile 1.6 mm ID Tygon tubing positioned in a BioRad EP-1 peristaltic pump (Bio-Rad Laboratories, Hercules, CA, USA). CSF was collected at a rate of 0.1 ml/minute into polypropylene tubes maintained in a refrigerated Biorad 2110 fraction collector. Initiation of CSF sample collection occurred approximately 30 and 10 minutes prior to and 0.5, 1, 2, 3, 4, 5, 6, 8, 10, and 12–14 hours following oral placebo administration. Individual samples were immediately aliquoted and frozen after each 2 ml sample was collected and stored frozen at –70 °C.

Lilly Study 7 was conducted at Washington University. Institutional review board approval was obtained from Washington University. The protocol conformed to the International Conference on Harmonization guidelines and to the Declaration of Helsinki. Written informed consent was obtained from all subjects. The study was a parallel, double-blind, randomized comparison of the effects of single oral doses of placebo and LY450139 on the rate of Aβ formation in seven healthy male volunteers aged 21–50 years (mean ± SD = 34 ± 7) [18]. CSF was collected via an indwelling subarachnoid catheter placed at the L3/L4 interspace. CSF sampling occurred at ~7:00 a.m. and continued hourly for 36 hours during and after intravenous administration of a stable isotope-labeled amino acid (^13^C_6_-leucine) as described previously [28, 29]. The oral placebo was administered to seven male subjects. CSF was frozen and maintained at –80 °C until analyzed.

Lilly Study 8 was conducted at PAREXEL International Early Phase (Los Angeles, CA, USA). Institutional review board approval was obtained from the California Institutional Review Board. The protocol conformed to the International Conference on Harmonization guidelines and to the Declaration of Helsinki. Written informed consent was obtained from all subjects. The study was an investigator-blind, placebo-controlled, randomized, single-dose design study of LY2811376 in healthy subjects [30]. Ten of 30 healthy subjects (27 males) aged 21–49 years (mean ± SD = 33 ± 8) participating in the CSF sampling portion of the study were administered placebo. The indwelling lumbar catheter was placed by anesthesiologists 4 hours before oral placebo administration. Subjects remained supine for the duration of the CSF sample collection period. Up to 22 CSF samples were collected at regular intervals, from 4 hours before up to 36 hours after study treatment administration (approximately –4, –2, 0, 1, 2, 3, 4, 5, 6, 7, 8, 9, 10, 12, 14, 16, 18, 20, 24, 28, 32, and 36 hours relative to dose administration). CSF samples were collected in polypropylene tubes, temporarily stored at –20 °C, aliquoted, and subsequently maintained at –70 °C until analysis.

#### Lilly analytical assay methods

For Lilly Study 1, Study 2, Study 3 and Study 6, CSF samples were analyzed at Eli Lilly and Company (Indianapolis, IN, USA) using proprietary 96-well plate-format enzyme-linked immunosorbent assays (ELISAs) developed and validated for measurement of Aβ42 and Aβ40_._ Coating of wells with capture monoclonal antibodies provided specificity for the C-terminal antigenic determinants of Aβ42 (antibody 21F12) and Aβ40 (antibody 2G3). The Aβ species recovered from CSF in the two assays were detected using a biotin-labeled monoclonal antibody raised to an Aβ N-terminal antigenic determinant (antibody 3D6). Concentrations of Aβ species were determined by measurement of biotin with horseradish peroxidase-labeled streptavidin and a colorimetric TMB substrate. The CSF Aβ42 ELISA provided a dynamic range of 20–250 pg/ml with intra-assay precision ranging from 2.67 to 3.06 (%CV), and inter-assay precision ranging from 3.61 to 5.28 (%CV). The CSF Aβ40 ELISA provided a dynamic range of 20–250 pg/ml with intra-assay precision ranging from 2.97 to 7.53 (%CV), and inter-assay precision ranging from 3.32 to 7.53 (%CV). CSF ELISAs determined during analysis of specimens collected from the clinical study were comparable with those observed during formal validation of the assays as listed above. For Lilly Study 4, Study 5, and Study 8, this resulted in slightly shifted and broader dynamic ranges corresponding to 25–400 pg/ml for both analytes. For Lilly Study 7, CSF Aβ42 and Aβ40 were measured at Washington University using minor modifications of the proprietary ELISAs methods described above.

### Methods from Merck serial sampling studies

The seven Merck studies were conducted at three sites, including Vanderbilt University (Nashville, TN, USA) PAREXEL/California Clinical Trials (Glendale, CA, USA) SGS Clinical Research (Antwerpen, Belgium). Institutional review board approval was obtained at each study site: Vanderbilt University, California Clinical Trials Medical Group, and SGS Clinical Research. The protocol conformed to the International Conference on Harmonization guidelines and to the Declaration of Helsinki. Written informed consent was obtained from all subjects. All participants were in good general health and without neurologic disease. After overnight fast, intra-dermal lidocaine anesthesia was applied prior to insertion of the spinal needle. Intravenous infusion of heparinized 5 % dextrose in water was performed to maintain hydration. A spinal needle was inserted through either the L2/L3 or the L3/L4 vertebral interspace, between 7:00 and 8:00 a.m. After entry into the subarachnoid space, the intrathecal catheter was secured externally with adhesive tape. In Studies 1 and 2, the catheter was extended with silicon tubing attached to a peristaltic pump with a continuous pump rate of 0.1 ml/minute for 24 hours in Study 1 and 0.5 ml/minute for the following 15 hours in Study 2. In Studies 3–7, a 5 ml syringe was attached to the intrathecal catheter and CSF was collected by gentle aspiration. The sampling volume and intervals are presented in Table 2. Each CSF sample, collected into polypropylene tubes at 4 °C, was frozen on dry ice within 30–60 minutes of collection for measurement of Aβ peptides. At the completion of sampling, the catheter was removed and the subject remained recumbent for 2 hours and then slowly and progressively moved to a sitting position over 8 hours. Subjects were observed for a minimum of 36 hours after cannula removal.

#### Sample collection and assay procedures

CSF samples from both Study 1 and Study 2 were analyzed for Aβ40 using an ELISA kit from Biosource International (Life Technologies, Grand Island, NY, USA) and for Aβ42 using an ELISA kit from Innogenetics NV (Ghent, Belgium). CSF samples from Studies 3–7 were analyzed for Aβ peptides using ELISA kits from Meso Scale Discovery (Gaithersburg, MD).

### Methods from Radboud University Medical Center serial sampling studies and assay procedures

The subject information and methods for serial CSF collection have been described previously [31]. Institutional review board approval was obtained from Radboud University Nijmegen Medical Center. The protocol conformed to the International Conference on Harmonization guidelines and to the Declaration of Helsinki. Written informed consent was obtained from all subjects. Six participants with mild AD (59–85 years, MMSE 16–26) and six healthy older volunteers (64–77 years) received an intrathecal catheter from which 6 ml CSF was collected every hour for 36 hours. Lumbar catheters were placed between 8:00 and 9:00 a.m. and CSF collection began at 10:00 a.m. Participant meals and schedules were standardized, but sleep was not. CSF aliquots were collected in polypropylene tubes and stored at −80 °C within 30 minutes of draw. CSF Aβ42 concentrations were determined using the xMAP-based INNO-BIA assay (Innogenetics NV) and CSF Aβ40 was determined using an ELISA (The Genetics Company, Schlieren, Switzerland) according to the manufacturer’s specifications. For validation of the results, CSF Aβ42 was repeated at Merck Research Laboratories (Kenilworth, NJ, USA) using the same assay, and CSF Aβ40 and Aβ42 were also repeated using a MSD multiplex assay Meso Scale Discovery (Gaithersburg, MD).

### Methods from Washington University serial sampling studies and assay procedures

Institutional review board approval was obtained from Washington University. The protocol conformed to the International Conference on Harmonization guidelines and to the Declaration of Helsinki. Written informed consent was obtained from all subjects. Fifty-seven subjects ranging from age 19 to 84 years were enrolled in the study. An intrathecal lumbar catheter and an intravenous catheter were placed between 7:30 and 9:00 a.m. and sample collection started between 8:00 and 9:30 a.m. for all participants. Six milliliters of CSF were obtained each hour for 36 hours. The CSF aliquots were frozen at −80 °C immediately after collection in 1.7 ml Axygen maximum-recovery polypropylene tubes (Corning, Corning, NY, USA). Participants were encouraged to stay in bed and were allowed free choice of when to sleep, read, watch television, use their laptops, or talk throughout the study. Analysis methods for Aβ40 and Aβ42 are as described previously [11].

### Method of data integration

The data were combined for an integrated analysis of the lumbar sample studies and from the indwelling catheter studies. Because each study and research group had different methods of sample collection, volume collected, and ELISAs, the data were transformed to a percentage of the mean in order to minimize the effects of the various extraneous factors. The data transformation was computed separately for Aβ40 and Aβ42.

It is well known that CSF amyloid concentrations are log-normally distributed [32] and all sample results were log-transformed. For the lumbar puncture results, the difference between the endpoint result and the baseline result was used as the primary response variable. For the repeated samples taken over 10–40 hours of indwelling catheter sampling, the concentrations were log-transformed and the mean concentration for each subject was computed. The difference from the mean concentration at each sampling time for each subject was computed. The differences were back-transformed and multiplied by 100 to obtain the percent concentration of the mean for each sample. The percent of the mean was used as the primary response variable.

All statistical programming and analysis was conducted in SAS version 9.1 or higher (SAS Institute Inc., Cary, NC, USA). The main statistical method used was the mixed procedure in SAS.

### Analysis of serial sampling during the initial 6 hours

To identify factors relevant to the CSF Aβ increase, CSF Aβ levels taken within the first 6 hours were analyzed for each individual. To identify differences in clinical trial design that may have affected Aβ levels, the data from Washington University was not included in this analysis because the same study design was employed for all of their studies and was the largest dataset (32 % of total participants). Owing to multiple observations from each subject, a linear mixed model was used to analyze the data only from the industry studies. The subject-specific random effects allow the model to account for the within-subject correlation. The response was log-transformed. The model included age group, number of draws within the first 6 hours, source/companies, and time as covariates. Except for time, all covariates were categorical in nature. There were three age groups defined as “young” (<30 years old), “medium” (between the ages of 30 and 50), and “old” (50 years and older). Similarly, the number of draws within the first 6 hours were also categorized into three levels (groups 1, 2, and 3): group 1 had three draws or less in the first 6 hours, group 2 had greater than three but less than seven draws in the first 6 hours, and group 3 subjects had seven draws or more within the first 6 hours. The data originated from four different sources. Additionally, an interaction effect between time and number of draws was considered in the model.

SAS 9.2 was used to analyze the data from the model specified above. During the analysis we appropriately choose a covariance structure. The unequal size of the response vector led us to select the SAS option “KenwardRoger” as a method for denominator degree of freedom. The interaction effect and the corresponding main effects came out as highly significant for both Aβ isoforms. The normality assumption was also verified through residual plots.

### Analysis of Washington University data

The data consisted of 392 observations from 57 subjects. The Aβ levels for the first 6 hours were used for the analysis. Both of the response variables (Aβ40 and Aβ42) were log-transformed to obtain sufficient symmetry in their distribution. Two age groups were created, based on whether a subject was under or over 65 years of age. Owing to the data being longitudinal I, a linear mixed model was most appropriate for analysis. The subjects were used as the block under repeated statement of mixed procedure of SAS to account for the correlation of the observations from the same subjects. The model included the age group and hours after first draw (including the 0 hour). The covariance structure was also chosen appropriately. The normality assumption was verified through residual plots.

### Analysis of CSF diurnal effects on Aβ

All participants from all sponsored studies using indwelling lumbar catheters were including for cosinor analysis. For each time point, Aβ concentrations for both Aβ40 and Aβ42 over time were expressed as the percent of the mean for each individual participant. Average percent of the mean for Aβ40 and Aβ42 was calculated at each time point for all of each sponsor’s participants. Owing to variability in study designs within individual sponsors, the number of samples collected at each time point is unequal. Cosinor analysis was performed similar to that described previously [10] except that the linear rise and cosine transformation were fitted simultaneously using the equation:

$$ \mathrm{Y} = \mathrm{amplitude}\ *\  \cos\ \left(\left(2*\mathrm{pi}\right)\left(\mathrm{X}/24\right) + \mathrm{acrophase}\right) + \left(\mathrm{slope}\ *\ \mathrm{X} + \mathrm{b}\right). $$

The cosine transformation was applied to the time variable using 24 hours as the default circadian cycle. Graphpad Prism version 6.0b for Mac (Graphpad Software, San Diego, CA, USA) was used to estimate the parameters of the circadian patterns for Aβ40 and Aβ42. The mesor (midline of the oscillation), amplitude (distance between the peak and mesor), and acrophase (the time corresponding to the peak of the curve) were calculated for each group. Note, the *y*-intercept, b, is equal to the mesor in the above equation.

## Results

### Indwelling catheter findings

#### Variability of Aβ concentration

**Fig. 1:**
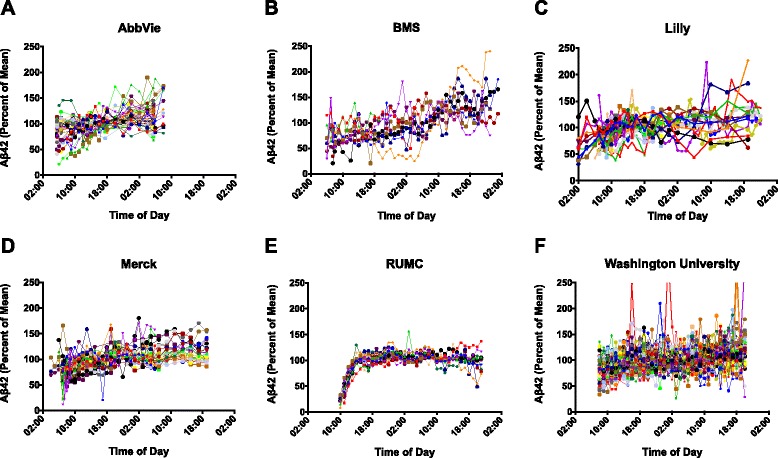
Profile of percent change of the mean in Aβ42 for each subject studied by each sponsor with serial sampling of CSF for up to 40 hours post catheter placement. Percent change of the mean is on the *y* axis and time of day for each study sponsor is shown on the *x* axis. **a** AbbVie Inc., **b** Bristol-Myers Squibb (BMS), **c** Eli Lilly and Company, **d** Merck and Company, **e** Radboud University Medical Center (RUMC), **f** Washington University

**Fig. 2:**
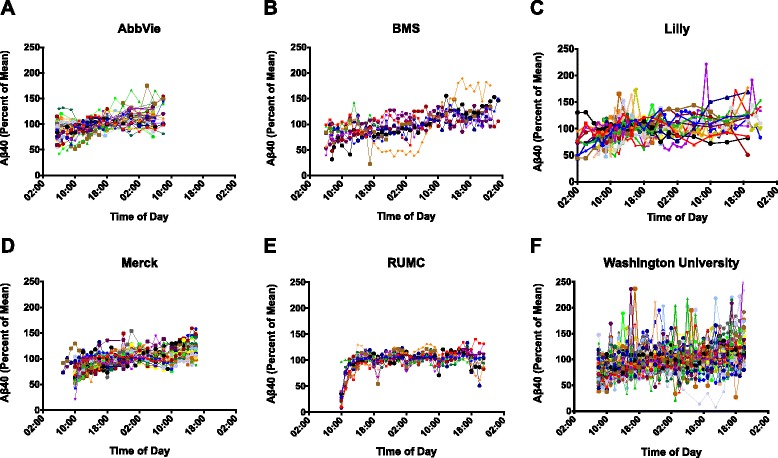
Profile of percent change of the mean in Aβ40 for each subject studied by each sponsor with serial sampling of CSF for up to 40 hours post catheter placement. Percent change of the mean is on the *y* axis and time of day for each study sponsor is shown on the *x* axis. **a** AbbVie Inc., **b** Bristol-Myers Squibb (BMS), **c** Eli Lilly and Company, **d** Merck and Company, **e** Radboud University Medical Center (RUMC), **f** Washington University

Variability in the concentration of Aβ40 and Aβ42 was found in the untreated and placebo-treated subjects enrolled in 16 separate serial CSF sampling studies (Table 2). Both Aβ42 and Aβ40 concentrations exhibited marked variability as a function of time (Figs. 1 and 2). A gradual upward drift was generally noted for both analytes and the observed increases did not return to baseline at 24 hours or even after extended sampling for as long as 36 hours.

A distinctly different pattern was observed in one serial CSF sampling study characterized by a sharp increase in Aβ40 and Aβ42 concentrations immediately after initiation of sample collection and followed by a rapidly achieved plateau that was sustained through the remaining sample collection (Figs. 1e and 2e). In this one study, a bacterial filter was incorporated into the sample collection device and was shown to interact with CSF Aβ40 and Aβ42 concentrations recovered from the sample matrix during the first 6 hours of collection, as reported previously [31]. As a result of this methodological difference, data from this one study (Radboud University Medical Center) were not included in all subsequent data analyses.

**Fig. 3:**
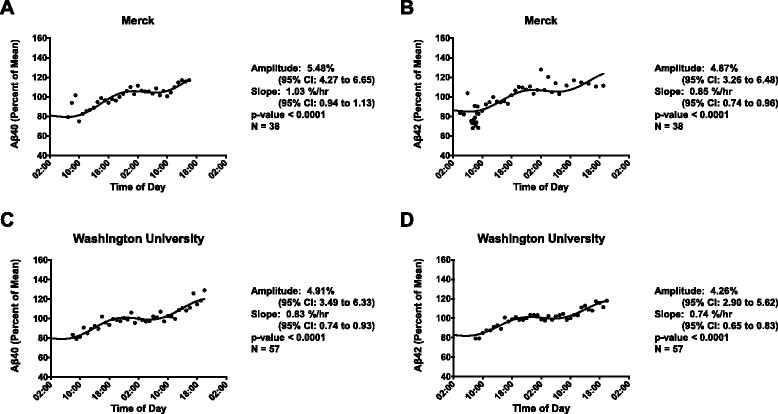
Diurnal oscillation of Aβ peptides in participants from two different study cohorts. Data presented as mean-adjusted average Aβ levels of the group over time of day for all subjects. Data from Merck and Company for (**a**) Aβ40 and (**b**) Aβ42, and data from Washington University for (**c**) Aβ40 and (**d**) Aβ42. Mesor-to-peak amplitudes of diurnal fluctuation of Aβ40 were 5.48 % for Merck and 4.91 % for Washington University. Mesor-to-peak amplitudes of diurnal fluctuation of Aβ42 were 4.87 % for Merck and 4.26 % for Washington University. The cosine transformation for all datasets was significantly different from a straight line (all *p* <0.0001). *CI* confidence interval

Previous work has shown that Aβ40 and Aβ42 concentrations vary with the sleep–wake cycle [10]. Cosinor analysis was used to compare circadian patterns of CSF Aβ dynamics from all of the studies with serial CSF sampling. The sleep–wake cycle was not monitored in these participants and could not be incorporated into the analysis. There were two sponsors with a large number of participants who underwent similar clinical trial designs in terms of frequency of CSF draws. The seven Merck studies (M1–M7) involved 38 participants (mean age 44.2 years, SD 11.2). The second study from Washington University included 57 subjects (mean age 60.7 years, SD 19). In both studies, CSF draws were taken approximately every 1–2 hours for 24–36 hours. Fig. 3 shows the simultaneous fit of the linear rise and cosine transformation to the mean-adjusted group average (percent of mean) data for the Merck and Washington University studies. Both Aβ40 and Aβ42 showed significant cosinor fits for both studies compared with a straight line (*p* <0.0001 for all analytes). Further, the 95 % confidence intervals for amplitude do not cross zero for both sets of studies (Fig. 3). The slope of the linear rise is also similar for both Aβ40 and Aβ42. The amplitude of the Merck studies is higher than that seen in the Washington University data, a difference possibly attributable to an overall younger population. Finally, the amplitude peaks occurred at approximately 20:00–21:00 p.m. in both studies and for both Aβ40 and Aβ42.

**Fig. 4:**
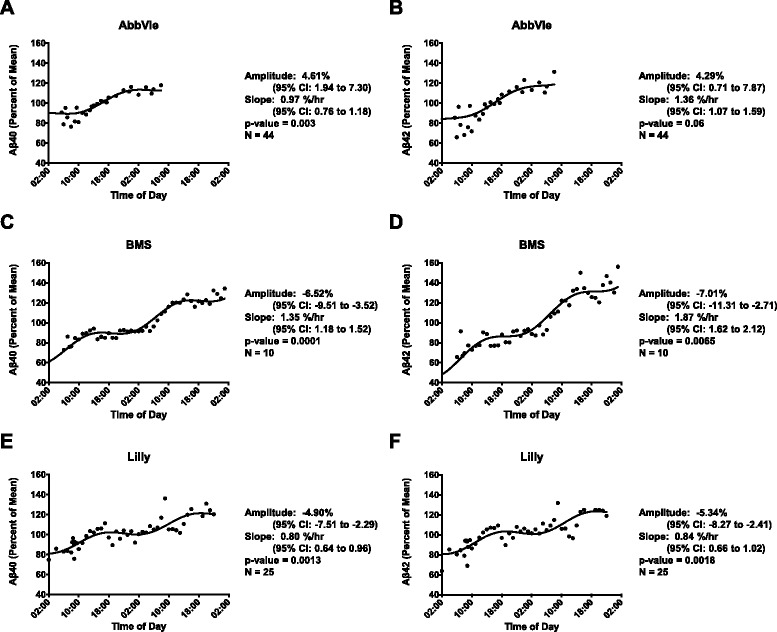
Diurnal oscillation of Aβ peptides in participants from three different study cohorts. Data presented as mean-adjusted average Aβ levels of the group over time of day for all subjects. Data from AbbVie Inc. for (**a**) Aβ40 and (**b**) Aβ42, from Bristol-Myers Squibb (BMS) for (**c**) Aβ40 and (**d**) Aβ42, and from Eli Lilly and Company for (**e**) Aβ40 and (**f**) Aβ42. Mesor-to-peak amplitudes of diurnal fluctuation of Aβ40 were 4.61 % (AbbVie), –6.52 % (BMS), and –4.90 % (Lilly). Mesor-to-peak amplitudes of diurnal fluctuation of Aβ42 were 4.29 % (AbbVie), –7.01 % (BMS), and –5.34 % (Lilly). The cosine transformation for all datasets were significantly different from a straight line except for Aβ42 from the AbbVie studies (*p* = 0.06). *CI* confidence interval

We also performed cosinor analysis on the mean-adjusted group average (percent of mean) data from three additional sponsors: AbbVie, BMS, and Lilly (Fig. 4). Despite variability in study design, numbers of participants, and sampling at each time point, studies from all sponsors showed a cosine fit for Aβ40 concentrations and all but AbbVie studies showed a cosine fit for Aβ42 concentrations. Peak amplitude showed greater variability in these three studies and occurred at different times of day, although the time was the same for Aβ40 and Aβ42 within each study sponsor. AbbVie studies had similar peak times for both Aβ40 and Aβ42 compared with the Merck and Washington University studies (21:00–22:00 p.m.). Both the Lilly and BMS studies had earlier peak times. For the Lilly studies, this may be due to an overall earlier start time: the Lilly studies began CSF collection as early as 02:00 a.m. and had a peak time of 16:00 p.m. for both Aβ40 and Aβ42. An earlier start time, however, does not account for the BMS studies that began CSF collection at 06:00 a.m. and had a peak time of 13:00–14:00 p.m. Differences in participant sleep–wake activity are unknown for all studies; therefore we cannot account for changes in sleep–wake patterns between studies that may correlate with differences in peak times. BMS was found to have the highest amplitude and slope for Aβ40 and Aβ42, but also had the youngest participants (mean age 31.6 years, SD 6.5) compared with Merck, Washington University, AbbVie (mean age 55.8 years, SD 17.8), or Lilly (mean age 42.4 years, SD 14.3) studies.

Please see Additional file 1 for the graphs from Figs. 3 and 4 with standard error of the mean intervals.

#### Linear rise in Aβ concentration

Statistical analysis was performed to identify factors that were significantly associated with the change in increasing linear trend for both Aβ40 and Aβ42 over time. We analyzed the slope of the Aβ increase over the first 6 hours relative to the number of CSF draws made during that time period. Since the studies differed in duration, only the first 6 hours were used in the analysis. The increase appeared to be the most dramatic during this time period. Factors such as age group, gender, ethnicity, frequency of draws, sponsor, and time were analyzed. For this analysis, subjects were divided into three age groups defined as “young” (<30 years old), “medium” (between the ages of 30 and 50), and “old” (50 years and older). Similarly, the number of draws within the first 6 hours were also categorized into three levels (groups 1, 2 and 3): group 1 had three draws or less in the first 6 hours, group 2 had greater than three but less than seven draws in the first 6 hours, and group 3 subjects had seven draws or more within the first 6 hours.

Of these factors, only the frequency of the CSF draw, time, and the interaction of draw frequency and time were significant (Table 3). The repeated-measures analysis (or linear mixed model), described in Materials and methods, showed that the interaction effects of the number of draws relative to time were significant for both Aβ40 and Aβ42. We further obtained the estimates of the slope of time within each draw group. All of the estimates were positively associated with the frequency of CSF draws. Further analysis revealed that the slope over time for draw groups 3 differed significantly from zero (Table 4), indicating that more frequent CSF draws resulted in a larger slope. Several factors were not known for the majority of the studies, most importantly amyloid status and sleep parameters that may affect the slope of the linear rise.

**Table 3:** ANOVA of fixed effects for industry in-dwelling catheter studies for Aβ40 and Aβ42

	Aβ40		Aβ42	
Effect	*F* value (df1, df2)	*p* value	*F* value (df1, df2)	*p* value
Age group	0.78 (2,156)	0.46	1.75 (2, 38.5)	0.19
Draw group	9.76 (2, 62.3)	0.0002*	11.07 (2, 15.1)	0.001*
Sponsor	0.94 (3, 137)	0.42	0.85 (3, 112)	0.47
Time	23.3 (1, 145)	<0.0001*	19.66 (1, 43.7)	<0.0001*
Time × draw group	11.55 (2, 139)	<0.0001*	8.5 (2, 22.3)	0.0018*

*ANOVA* analysis of variance, *df* degrees of freedom

*p < 0.05

**Table 4:** Slope and significance for time × draw groups 1–3 interaction for Aβ40 and Aβ42

	Aβ40		Aβ42	
Time × draw group	Slope	*p* value	Slope	*p* value
1	0.41	0.56	1.02	0.25
2	1.6	0.12	0.79	0.45
3	4.12	<0.001*	4.32	<0.001*

*p < 0.05

Age was not a significant factor for the combined data. In the industry studies, however, only 15 out of the 107 subjects (minus the RUMC data) were older than 65 years. In contrast, 36 out of 57 subjects in the Washington University dataset were older than 65 years. Analysis of this dataset alone showed a trend towards an effect of age on the rise in Aβ levels in the first 6 hours. Younger subjects showed a greater change in Aβ42 levels over time relative to older subjects (*p* = 0.02); however, for Aβ40 there was no significant difference between the age groups in terms of the change in Aβ levels in the first 6 hours (*p* value = 0.17). Again, this difference between age groups and Aβ isoforms may be due to amyloid deposition, a factor not included in this analysis.

### Lumbar puncture findings

**Fig. 5:**
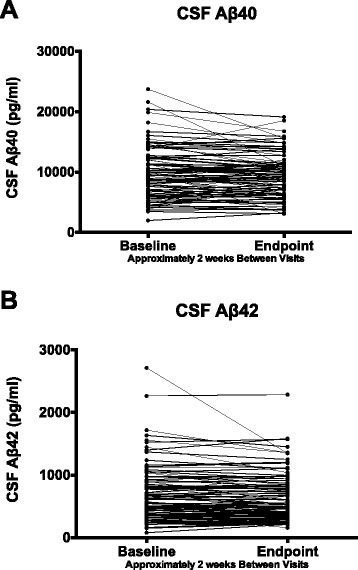
Profile plot of cerebrospinal fluid (CSF) (**a**) Aβ40 and (**b**) Aβ 42 from subjects in Lilly lumbar puncture studies given placebo for approximately 2 weeks with lumbar punctures obtained at baseline and endpoint

For untreated or placebo-treated subjects enrolled in four separate clinical studies, intra-subject variability for CSF Aβ40 and Aβ42 collected by lumbar puncture were generally stable on comparison between baseline and endpoint sampling scheduled approximately 2 weeks apart. Occasionally, individuals exhibited notable shifts from baseline (increase or decrease); however, the majority of subjects exhibited stable results. Within individual studies, lumbar punctures were performed at approximately the same time of day. Overall, no significance difference between the endpoint and baseline for Aβ42 and Aβ40 was observed (*p* = 0.54 and *p* = 0.14 respectively) (Fig. 5a,b). In addition, Aβ40 and Aβ42 results for individual samples were highly correlated (*r* = 0.8; *p* <0.001).

## Discussion

We examined CSF Aβ40 and Aβ42 concentrations across multiple studies from four different companies and two academic institutions. While a tremendous amount of inter-individual variability existed, a general pattern of rapidly increasing concentrations of Aβ40 and Aβ42 in the first 12 hours of a study was found across all subjects in all indwelling lumbar catheter studies; in some cases, Aβ40 and Aβ42 reached levels as high as 200 % from the initial baseline draw. In most of the subjects, the slope of the linear rise decreased after the first 24 hours, but the levels did not return to baseline by 24 hours. Our results suggest that the use of indwelling catheters to collect multiple CSF samples results in an upward drift in the levels of both Aβ40 and Aβ42 relative to the initial draw. In contrast, the levels of Aβ40 and Aβ42 did not change significantly from baseline to second draw when samples were taken by lumbar puncture performed at a similar time of the day 2 weeks apart.

The tremendous inter-subject variability of Aβ40 and Aβ42 seen over time could influence the results of AD prevention studies that target Aβ. Averaging Aβ concentrations at each time point is recommended to minimize the effect of individual variability and distinguish effects between experimental groups. We further recommend managing this variability by calculating the area under the curve using pharmacokinetic–pharmacodynamic models. For example, a previous study of a gamma-secretase inhibitor was able to show relatively small effects on Aβ in CSF collected via indwelling lumbar catheters using this technique [18].

A circadian rhythm is a cyclic oscillation intrinsic to many biologic processes, such as body temperature and hormone levels, and is typically studied using cosinor analysis to fit a cosine transformation with a 24-hour period. Aβ concentrations have been found to fluctuate over time in several studies collecting CSF via a lumbar catheter [8, 9]. Further, a previous study correlated fluctuating CSF Aβ levels to the sleep–wake cycle (i.e. a diurnal pattern) in healthy young normal control subjects and older adults more than 60 years old with and without amyloid deposition [10]. In that study, younger controls demonstrated much higher amplitude of Aβ oscillation as compared with amyloid-positive or amyloid-negative older adults.

Despite heterogeneity in the frequency of CSF collection in this study, we found evidence in data from all sponsors for a diurnal pattern of both Aβ40 and Aβ42. The only exception was the AbbVie data for Aβ42 which showed a mesor-to-peak amplitude of 4.29 % with 95 % confidence intervals that do not cross zero (0.71–7.87) suggesting a cosinor fit, but only a trend toward a difference with a straight line was noted (*p* = 0.06). This discrepancy is attributable to both the scatter of the data points in the first and last 6 hours of sampling due to CSF collection occurring at slightly different times of day across the AbbVie studies and a limited sampling time of 24 hours, the shortest period among all of the sponsors. Prior work analyzed a smaller group of subjects from the AbbVie studies (*n* = 21 compared with *n* = 36 in our analysis) and reported that intra-subject variability was related to sampling frequency and/or sampling volume [12]. We suspect a diurnal oscillation was not seen in this prior work because each study was analyzed separately (*n* = 6–8/study) and not as a larger pooled analysis.

Cosinor parameters, such as amplitude, differed across all studies. Aβ40 and Aβ42 showed similar oscillations within study sponsor, but not between all study sponsors. The peak-to-trough amplitude fluctuations of Aβ40 and Aβ42 varied between sponsors from approximately 8.52 to 14.02 % per 24 hours. The peak time of Aβ levels also varied between study sponsors. For example, both the Merck and Washington University datasets were found to have Aβ levels peak at roughly 13–14 hours after the collection was started (which is equivalent to ~20:00–21:00 p.m.). BMS, however, peaked earlier for both Aβ40 and Aβ42 at approximately 8 hours after collection started (14:00 p.m.). In contrast, Aβ40 and Aβ42 peaked at approximately 14 hours after collection started in the Lilly studies (16:00 p.m.) and at approximately 16–17 hours after collection started in the AbbVie studies (21:00–22:00 p.m.).

These discrepancies in the amplitudes and peak time of CSF Aβ fluctuation are possibly due to differences in participant ages, sample collection schedules, analytic techniques used between sites, participant stress levels, participant amyloid status, and differences in sleep–wake activity between participants in the different groups. Our findings suggest that the Aβ diurnal pattern is not a timekeeper that directly interacts with the circadian clock, but is dependent on human processes with a diurnal pattern—such as changes in neuronal activity with the sleep–wake cycle as described previously [10, 33]—and may be modifiable [34]. The timing and amount of participant sleep as well as light exposure were not monitored in these studies; therefore we cannot test our hypothesis that differences in sleep–wake activity accounts for the inter-study difference in Aβ oscillation. However, the finding of an Aβ diurnal pattern for both Aβ40 and Aβ42 despite study heterogeneity and other confounders strongly suggests that this oscillation is due to an intrinsic human biological process rather than an artifact of experimental design.

Since the studies enrolled subjects of different demographics and different protocols were employed for CSF collection across the studies, statistical analysis was conducted to identify factors that were associated with the rise in Aβ levels. Factors such as age, gender, clinical sponsor, and frequency of CSF draws were all analyzed. In the industry-sponsored studies, where considerable variability existed in the CSF collection designs, the frequency of CSF draws was significantly associated with the Aβ rise. The results indicated that performing more than seven CSF draws in the first 6 hours of the study had a large effect on the rise in CSF Aβ, while the rise in CSF Aβ was largely not evident in studies that collected six or less draws in the first 6 hours. This finding is similar to previous reports from AbbVie that sampling frequency was a factor in the Aβ linear rise [12]. Several of the same studies sponsored by AbbVie are included in this analysis, but our study includes additional industry and academic sponsors and shows the same effect.

The Aβ linear rise is not a diurnal effect and is most probably a consequence of frequent CSF draws via an indwelling lumbar catheter leading to a shift in the rostrocaudal dynamics in CSF. A recent study showed that levels of Aβ were higher in CSF collected from the cisterna in patients suffering from trigeminal neuralgia relative to CSF from lumbar draws taken from patients suffering from tension-type headaches [35]. Potentially, frequent CSF draws from the lumbar region could result in an increase in the flow of CSF from the cisterna to the lumbar region, where Aβ levels may be higher. The volume of CSF collected may also be involved, although we could not address this question because all but one study (Study M3, *n* = 6; Table 2) collected 6–8 ml/draw. Factors that affect the concentration of Aβ40 and Aβ42 may also be involved in the linear rise. For instance, previous work has shown attenuation in the linear rise of Aβ42 in amyloid-positive individuals [10]. Studies that involve different CSF collection protocols and labeling of CSF Aβ may help to address this question [36].

The studies conducted at Washington University in St Louis enrolled subjects in different age groups. In these studies, there was a trend towards age being a significant factor for the rise in CSF Aβ levels, with younger subjects showing significantly higher increases relative to older subjects for Aβ42. Previous studies examining age and the rise in CSF Aβ levels did not show an effect in amyloid-negative subjects [10]. However, in that study, the rise in Aβ was calculated over a 24-hour period, rather than the first 6 hours. In addition, subjects that were amyloid-positive did display smaller increases in CSF Aβ levels over time.

### Implications for future clinical trial design

The issue concerning whether or not significant fluctuations in CSF Aβ concentrations occur owing to CSF collection methodology is of paramount importance for the design of clinical trials where these biomarkers would be utilized to study pharmacodynamic activity, and ultimately may determine whether these biomarkers have utility in a diagnostic fashion. Further, the use of a filter system in the sampling setup employed by RUMC illustrates that the selection of sampling materials and the configuration of those materials on the recovery of Aβ species from CSF pulled through catheter systems needs to be carefully evaluated. Overall, our analysis has identified that frequency of CSF draws and age are factors that contribute to the linear rise in Aβ levels. The Aβ diurnal pattern, in contrast, is suggested to be an intrinsic process that occurs independent of CSF collection methodology. These findings are important for the design of studies of Aβ. For instance, a recent trial investigated whether sleep deprivation resulted in an increase in CSF Aβ concentration [34]. The study did show an increase in the Aβ42 concentration in the sleep-deprived group compared with the normal sleep control group, but Aβ40 did not show the same pattern as Aβ42. This is in contrast to our results that show Aβ40 and Aβ42 oscillating together. The sleep-deprived group had an additional four CSF collection time points between hours 5 and 13 that complicate the study interpretation because differences in CSF collection may account for the rise in CSF Aβ42 concentration and discordance with Aβ40, although additional analyses of total protein showed that there was no effect of the differential sampling on CSF protein concentration between the two groups.

From our analysis, it is recommended that the frequency of CSF draws should kept to no more than 1/hour with a volume of 6–8 ml in order to minimize changes in CSF Aβ with frequent draws, particularly in the first 6 hours of collection; also, it is critical that a standard draw frequency be utilized across all study groups. Our data are not sufficient to determine whether these are the optimal CSF draw volume and frequency to control for the linear rise. Further, the linear rise needs to be accounted for during analysis of Aβ levels in CSF collected by indwelling lumbar catheters either by fitting both a cosine function and linear rise simultaneously as we have done, or by subtracting out the linear rise before cosinor analysis as reported previously [10].

## Conclusion

Indwelling lumbar catheters are an invaluable research tool for following changes in CSF Aβ over 24–48 hours, but factors affecting Aβ concentration such as linear rise and diurnal variation need to be accounted for in planning study designs.

## Additional file

Additional file 1: Figure S1. Shows plots of cosinor analysis of data from all sponsors as in Figs. 3 and 4 with standard error of the mean intervals shown.

## Abbreviations

Aβ: Amyloid-beta
AD: Alzheimer’s disease
ADNI: Alzheimer’s Disease Neuroimaging Initiative
BMS: Bristol-Myers Squibb
CSF: Cerebrospinal fluid
CV: Coefficient of variation
ELISA: Enzyme-linked immunosorbent assay
HPLC: High-performance liquid chromatography
MMSE: Mini-Mental State Examination
MRI: Magnetic resonance imaging
PET: Positron emission tomography
PiB: Pittsburgh Compound B
QC: Quality control check
RUMC: Radboud University Medical Center
SD: Standard deviation
