# Supplementary figures and images for: An integrated multi-study analysis of intra-subject variability in cerebrospinal fluid amyloid-β concentrations collected by lumbar puncture and indwelling lumbar catheter
Source: Alzheimers Res Ther. 2015 Jul 29;7(1):53. doi: 10.1186/s13195-015-0136-z (PMC4518529; doi:10.1186/s13195-015-0136-z)

## Slide 1
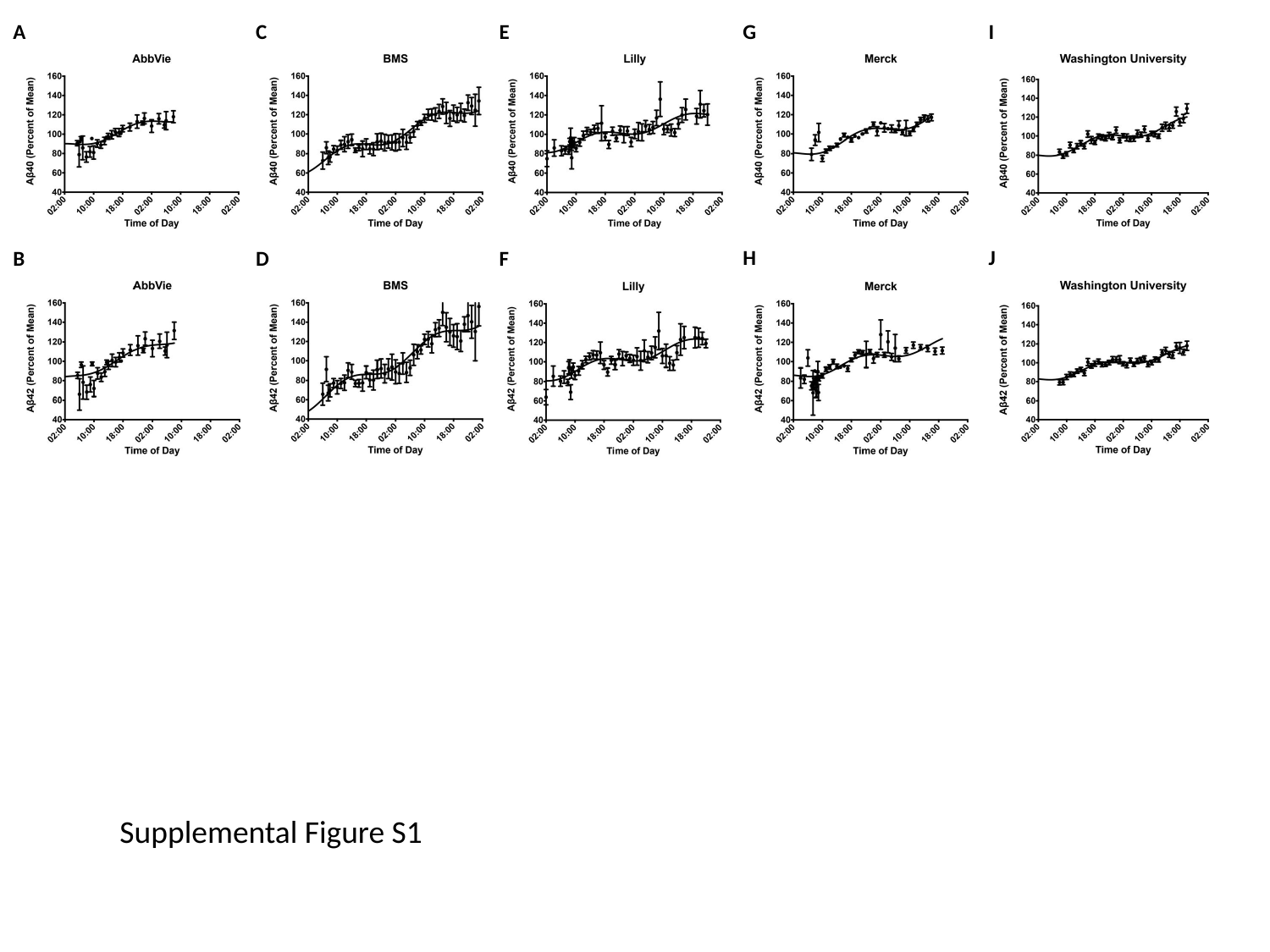

G
I
A
C
E
H
J
B
D
F
Supplemental Figure S1

Supplement: Additional file 1: Figure S1. — Shows plots of cosinor analysis of data from all sponsors as in Figs. 3 and 4 with standard error of the mean intervals shown. [file 13195_2015_136_MOESM1_ESM.pptx]
